# Tradition and trade: culture and exploitation of avian fauna by a rural community surrounding protected areas in the south of Bahia’s State, Northeastern Brazil

**DOI:** 10.1186/s13002-022-00515-x

**Published:** 2022-03-08

**Authors:** Antonio Iderval Sodré Neto, Ricardo Evangelista Fraga, Alexandre Schiavetti

**Affiliations:** 1 Programa de Pós-Graduação em Ecologia e Conservação da Biodiversidade – PPGECB, Universidade Estadual de Santa Cruz -UESC, Pavilhão Prof. Max de Menezes, 1º andar, sala 1DA., Rodovia Jorge Amado, km 16 - Salobrinho, CEP 45662-900 Ilhéus, Bahia, Brazil; 2 Instituto Multidisciplinar em Saúde, Campus Anísio Teixeira, Universidade Federal da Bahia, Rua Rio de Contas, 58, Candeias, Vitória da Conquista, BA 45029-094 Brazil; 3 Laboratório de Etnoconservação e Áreas Protegidas, Universidade Estadual de Santa Cruz, Departamento de Ciências Agrárias e Ambientais, Rodovia Jorge Amado km 16, Salobrinho, BR 45662-900 Ilhéus, Bahia, Brazil

**Keywords:** Ethnozoology, Protected areas, Wild bird trade, Bird-keeping

## Abstract

**Background:**

Illegal capture and trade of wild birds are some of the most present types of wildlife trade in Brazil, and are often associated with cultural and socioenvironmental aspects. Those habits are particularly present in rural communities, where bird trade can be a source of income in dire economic situations and bird-keeping is a cultural trait passed down from generations.

**Methods:**

We conducted a series of semi-structured interviews with bird-keepers and traders within the surrounding region of the *Parque Nacional de Boa Nova*, inquiring about local customs and practices related to bird-keeping, bird trade and bird capture, as well as how these were affected by the establishment of protected areas nearby. We then outlined the main trends and perceptions in a quantitative and a qualitative approach.

**Results:**

A total of 21 avian species were mentioned as being used as pets and in commercialization, contests and breeding, most of them occurring naturally in the region. Most respondents were men possessing low levels of education and income. We observed a series of specialized practices regarding bird-keeping, from basic maintenance of captive individuals in order to ensure the animal’s health, to interspecies breeding as to produce hybrid individuals. Mentioned methods used to capture wild birds often involved specialized traps and were conducted mainly within the national park’s area. Bird trade was said to occur mostly in urban settlements, and the value of captive birds was said to vary, based on species and beforehand training. The official establishment of the protected area impaired all practices related to bird-keeping and trade, mostly as a result of increased surveillance by environmental agencies.

**Conclusion:**

The collected information presents a series of specialized habits and practices involved in bird-keeping, bird capture and bird trade, many of them being associated with the local avifauna surrounding the region. The establishment of protected areas affected local perceptions regarding bird-keeping and related practices mostly through fear of penalty, although individuals demonstrated some knowledge about how to evade surveillance. We recommend further studies about effective ways to integrate local communities in nearby protected areas’ conservation.

## Background

The worldwide interest in wildlife products is assumed to be worth billions of US dollars every year; a fact that is especially relevant in poor countries and regions presenting considerable preserved remnants of natural resources, as those could be exploited by local residents as a potential alternative source of income amidst critical economic positions and an increasing demand for wildlife products [1–4]. One of the most popular instances of wildlife trade is the illegal commercialization of avian species; an operation that is especially relevant to South American countries, as those areas are often featured as sources and destinations for the international bird trade [5, 6]. The usage of wild avian species as pets is one of the main drivers for this illegal market [7], a prospect that creates demand for particular avian species and imposes pressure on natural populations, leading to population declines and extinctions globally [8–11].

In Brazil, the illegal trade and the captivity of avian species are practices often associated with economical vulnerability [3, 12], cultural aspects [13–15] and proximity to natural resources and conservation areas [16]. The main source of supply for the illegal bird trade often relates to rural settlements and communities, where local dwellers would be primarily responsible for capturing valuable species and selling them to middlemen for a fraction of the price that captive birds would reach in other locations such as big city centers [17]. The primary motivation for this interaction is described as economic vulnerability, as rural residents would see in the illegal bird trade an easy means of alternative income [18, 19]. Socioeconomical factors also seems to influence illegal bird trade activity at a municipal scale, as counties presenting higher socioeconomic metrics such as gross domestic product (GDP) and human development index (HDI) would be most associated with avian species commercialization, whereas municipalities presenting lower values of those metrics would be more related to bird capture and trapping, serving as source areas to supply the illegal demand for avian species [3]. At a demographic level, the profile of individuals who engage in bird-keeping and related activities is often associated with educational levels [20], gender [21] and income [22], and the commercialization of avian species is generally linked to economic vulnerability or a way to supplement income [23].

The relationship between socioenvironmental elements and wild bird trade is especially prevalent in the Northeast of Brazil, a region that is thought to shelter most of the source areas for wild bird capture and traffic [16–18]. In this region, illegal trade and transportation of wild birds is widely described occurring along main highways that connect many big city centers with small municipalities, effectively creating a trafficking route for illegal trade of avian species [16, 18, 23]. In fact, the relevance of roads and highways in the Northeast is so paramount to the wild bird trade that it often occurs on the very roadside of some main highways that connect this region to others in the country [19]. The vulnerability of protected areas to illegal wildlife hunting is another socioenvironmental issue that surrounds the wild bird trade, as the higher diversity of native species comprehended by these sites are often targeted by hunters [16, 24].

Although there are a number of works tackling the wild bird trade in Brazil at a broader scale [16, 17, 23], there is still a need to explore this theme from an ethnozoological point of view in order to reach a better understanding of wild bird trade implications and motivations [16, 25]. In this instrumental case study, we conducted a series of semi-structured interviews with local residents of a rural settlement adjacent to a federal protected area located close to two federal highways connecting the Northeast to other Brazilian regions; a site that is often described as a source area for supplying the wild bird trade in the region [19, 26–28]. In order to (i) identify the local ecological knowledge associated with bird-keeping and related practices, both as a hobby and a source of income, (ii) evidence corresponding demographic traits between bird-keepers and traders, and (iii) inspect the consequences of an protected area’s establishment on an local community’s ethnozoological perceptions, we conducted a series of qualitative and quantitative analysis highlighting the main species, habits, perceptions and socioenvironmental aspects related with bird-keeping, bird-capturing and bird trade. The observations presented here could help characterize the dynamics involved in bird trade and clarify how much bird-keeping is intertwined with socioenvironmental and cultural aspects of a region adjacent to a protected area known for its avian species diversity [29].

## Methods

### Ethical approval

All the individuals who participated in this research signed a consent form agreeing to be a participant beforehand, under the arrangement that no personal information that could reveal the participant’s identity or expose themselves to damage would be disclosed. This study was approved by the Research Ethics Committee of the Instituto Multidisciplinary em Saúde-Campus Anísio Teixeira under the CAAE no. 43546621.5.0000.5556.

### Study area

**Fig. 1:**
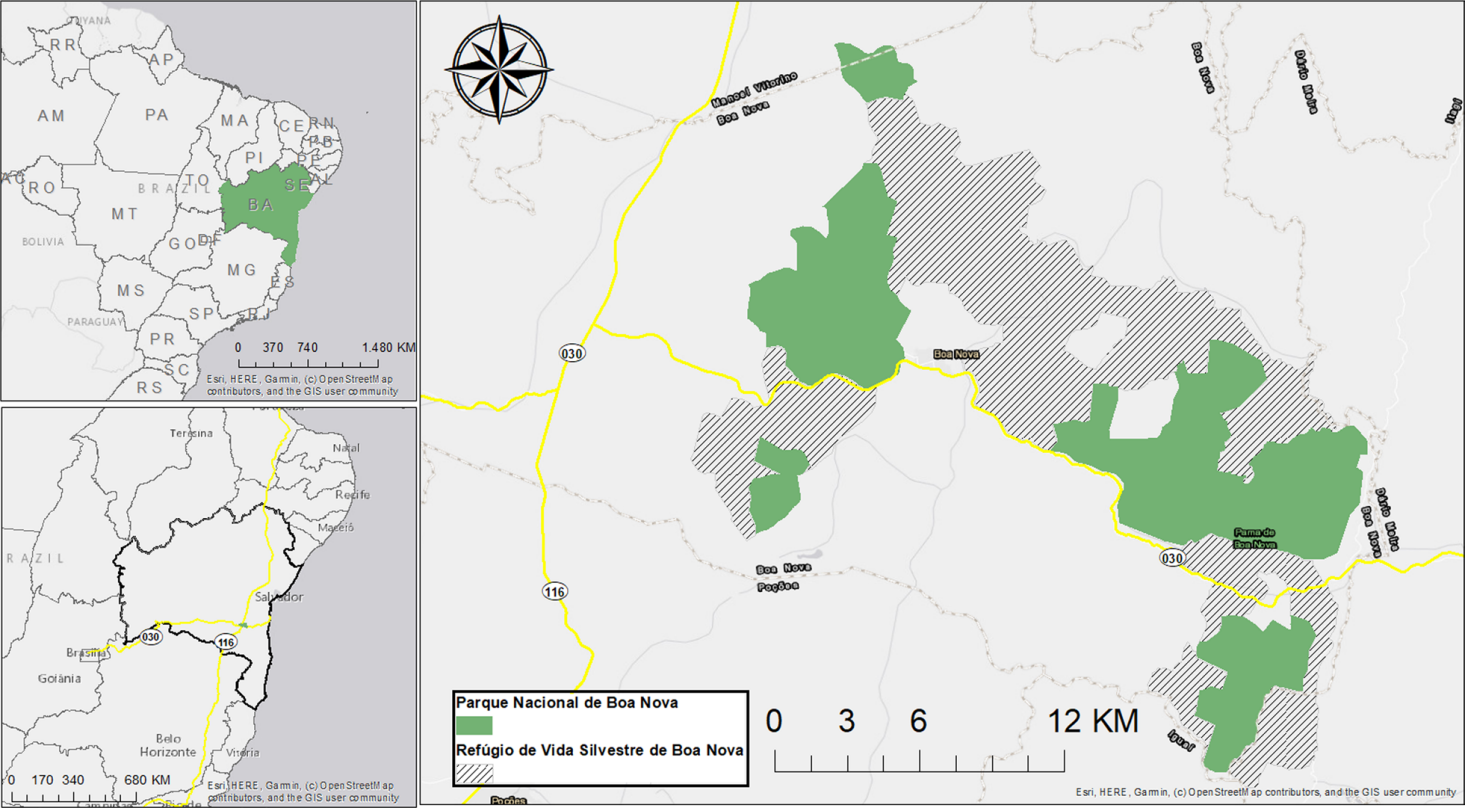
Study area. Location of the protected areas relative to the city of Boa Nova and nearby municipalities, as well as the highways BR-030 and BR-116

Our research was conducted within the rural and urban settlements of Boa Nova (Fig. 1), a municipality with an estimated population of approximately 12 thousand inhabitants. The main activities leading the local economy are related to agriculture and cattle raising, and only 6.7% of the population occupy a formal profession [30]. The city has a reputation of being a “paradise for birds”, with over 450 avian species being registered within the city’s surroundings, including endemic and migratory species [31–33]. As such, a local culture has been developed around this high avian diversity, and recently, protected areas were established in order to manage and conserve it [34].

The *Parque Nacional de Boa Nova* (cat II from IUCN) was established in 2010 to preserve the natural Atlantic Forest and Caatinga ecosystems located within the region, as well as the transition zone between those two known as “vine forest”. Alongside the park’s foundation, a buffer zone known as *Refúgio de Vida Silvestre de Boa Nova* (cat V from IUCN) was also established to serve as a link between the distinct fragments of the park [34]. Given the recent attention provided by the establishment of the protected areas, Boa Nova has been being developed as a tourist destination by official environmental agencies and nonprofit organizations, who also promote initiatives to integrate the local community on avian species conservation [29].

Two federal highways surround the protected areas: BR-030 and BR-116. Those are responsible for connecting the Northeast region with other regions in Brazil and are described as key points to local wild bird trade dynamics, often relating to the transport of newly-captured birds within the region to other places in the country, where those would be sold [19].

### Data collection and analysis

We used direct semi-structured interviews [35] to infer bird keeper’s local ecological knowledge of bird-keeping, capture and trade in the considered region, as well as personal perceptions about how those activities were affected after the establishment of the national park. To identify and select informants we used snowball sampling, a method where respondents ought to provide contact information about other potential informants that would be coherent to the research’s goal [36], as well as opportunistic sampling when feasible.

Qualitative data were treated by codification and triangulation, where relevant information was identified, compared, categorized and interpreted, enabling the classification of pertinent mentions and quotations into a larger cohesive context [37]. Quantitative analysis was conducted by calculating the use value ($UV$) relative to each cited species using a simplified formula derived from the original method proposed by Phillips & Gentry (1993): $UV = \sum U_{i} /n$; where $U_{i}$ = the number of uses stated by each informant for a particular species and $n$ = the total number of informants [38]. Intending to inspect the relationship between socioeconomic factors and practices involved in bird-keeping and commercialization, we conducted a Multiple Correspondence Analysis (MCA): an exploratory multivariate analysis, which summarizes the relationship between variables in a two-dimensional coordinate map where variables allocated closer to each other are more related [39]. In order to guarantee a higher quality of representation in our MCA, we filtered our results to exhibit only variables, presenting a *squared cosine* (*cos2*) higher than 0.4. We also created a chord diagram delving into the different uses attributed to each species, as well as the frequency of citations linking each species to each particular usage.

### Species identification

Species were identified through direct visualization and photographic records. Visual identification was done by comparing photographs with a Brazilian avian species identification guide [40]. Identification by popular name was done by comparing recorded citations with specifications from the Brazilian Ornithological Records Committee [41]. To confirm if mentioned species were coherent to the considered region, we consulted specific guide books concerning avian species occurring within the Atlantic Forest [42] as well as available records of avian species reported within the considered region, including online photographic registries [32, 33, 43]. The conservation status of cited species was determined by the guidelines of IUCN’s Red List [44].

## Results

### Bird-keepers’ socioeconomic profile

**Fig. 2:**
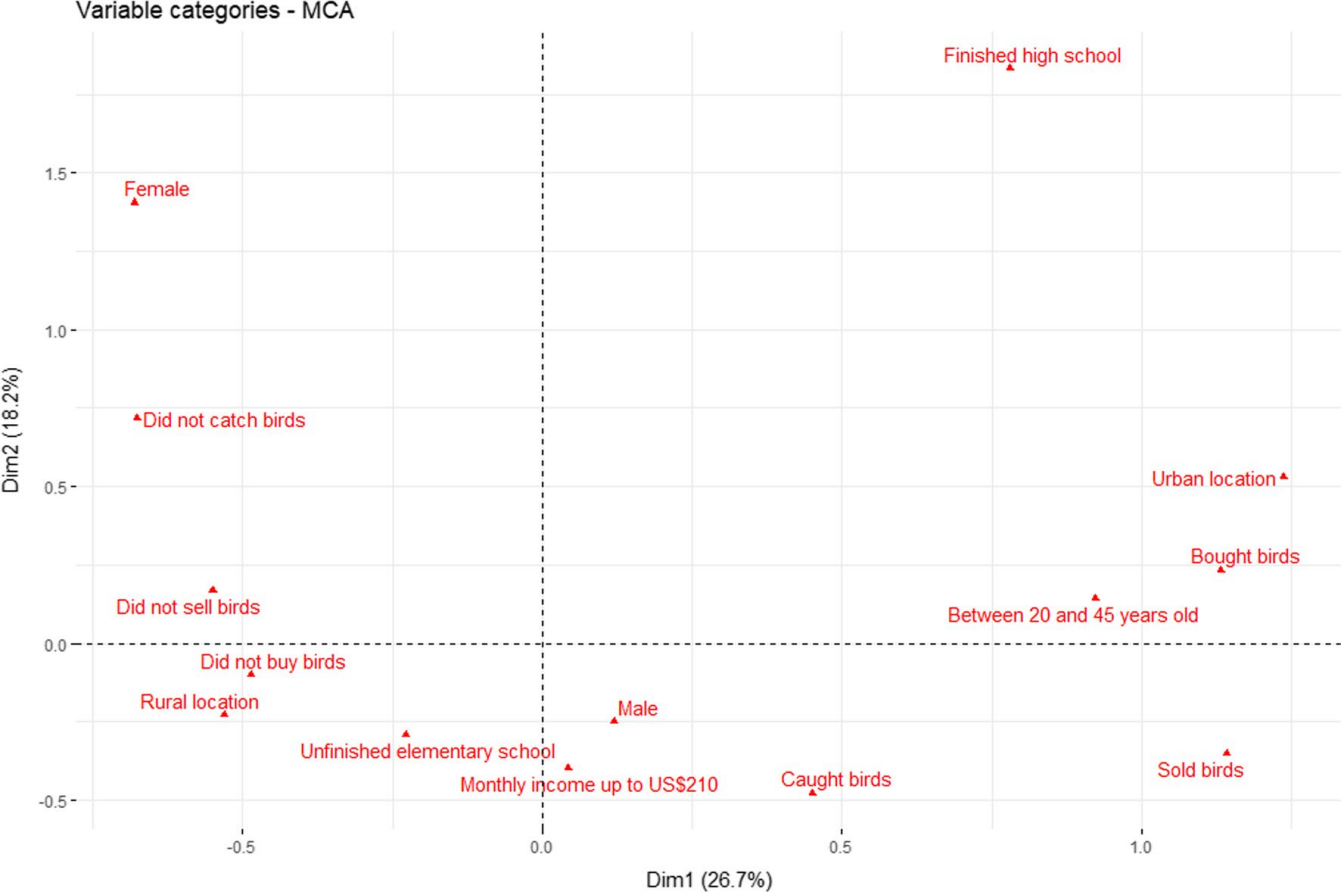
Multiple Correspondence Analysis displaying the relationship of analyzed variables. In this two-dimensional coordinate map, socioeconomic factors allocated closer to each-other suggest an association between analyzed variables. The presented information regarding capture, keeping and trade refer to the last year

We interviewed a total of 40 bird-keepers or ex-bird-keepers in the considered region. The majority of interviewed bird-keepers were men (n = 34), inhabited rural areas (n = 28), had only completed primary education (n = 32) and reported an income less than minimum wage (n = 29). Respondent’s age ranged from 20 to 78 years, with the mean being 48 years old. Our Multiple Correspondence Analysis points to a correlation between settlements and wild birds’ commercialization (Fig. 2). The majority of respondents that reported buying and selling wild birds inhabited the urban area and were from a specific age range (20 to 45 years old).

### Species and uses

**Fig. 3:**
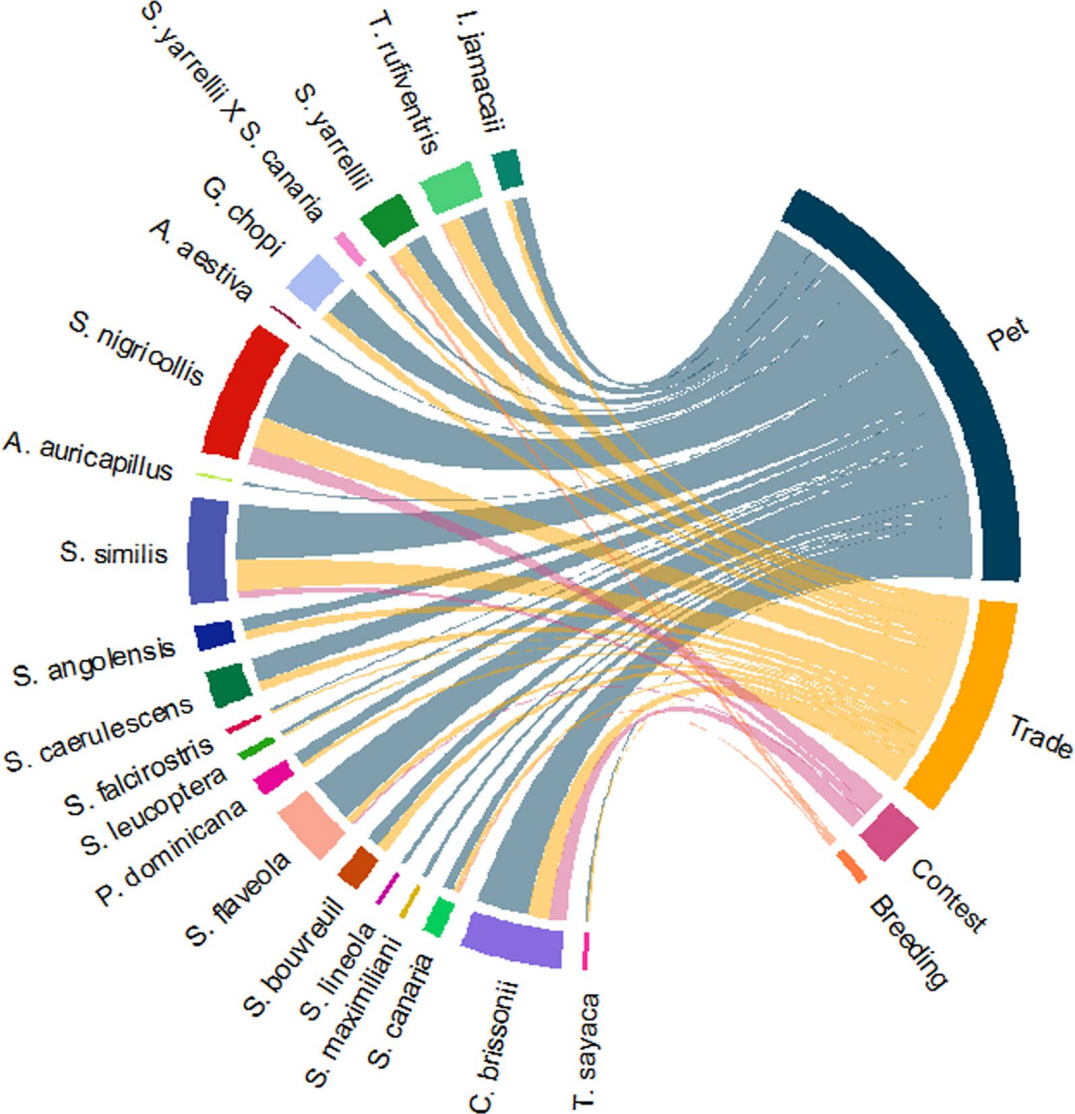
Chord diagram depicting frequency of usage attributed to each mentioned species. In this figure, each line associated with a species portrays one respective mention

A total of 21 avian species (including one hybrid) was mentioned, most of those being of the Passeriformes order (n = 19) and the Thraupidae family (n = 12). Four major uses were associated with birds: pet, trade, contest and breeding (Table 1). While keeping birds as pets was a practice associated with all mentioned species, trade was linked mainly to songbirds. Breeding and usage in the contests were related only to a few particular species (Fig. 3).

**Table 1:** List of mentioned species and number of citations regarding each type of use

Taxonomic categories (order/family/species)	Popular name	Citations per type of use				UV	Conservation Status (IUCN)
		Trade	Breeding	Pet	Contest		
Passeriformes							
Thraupidae							
*Sporophila nigricollis* (Vieiloot, 1823)	Yellow-bellied Seedeater	13	0	30	7	1.250	LC
*Saltator similis* (d'Orbigny & Lafresnaye, 1837)	Green-winged Saltator	13	0	23	3	0.975	LC
*Sicalis flaveola* (Linnaeus, 1766)	Saffron Finch	3	0	19	1	0.575	LC
*Sporophila caerulescens* (Vieillot, 1823)	Double-collared Seedeater	4	0	10	0	0.350	LC
*Sporophila bouvreuil* (Statius Muller, 1776)	Copper Seedeater	4	0	5	0	0.225	LC
*Sporophila angolensis* (Linnaeus, 1766)	Chestnut-bellied Seed-Finch	4	0	5	0	0.225	LC
*Paroaria dominicana* (Linnaeus, 1758)	Red-cowled Cardinal	2	0	5	0	0.175	LC
*Sporophila leucoptera* (Vieillot, 1817)	White-bellied Seedeater	1	0	2	0	0.075	LC
*Sporophila maximiliani* (Cabanis,1851)	Great-billed Seed-Finch	0	0	2	0	0.050	EN
*Sporophila lineola* (Linnaeus, 1758)	Lined Seedeater	0	0	2	0	0.050	LC
*Sporophila falcirostris* (Temminck, 1820)	Temminck's Seedeater	1	0	1	0	0.050	VU
*Tangara sayaca* (Linnaeus, 1766)	Sayaca Tanager	1	0	1	0	0.050	LC
Fringillidae							
*Spinus yarrellii* (Vieillot, 1805)	Yellow-faced Siskin	7	2	8	0	0.425	VU
*Serinus canaria* (Linnaeus, 1758)	Atlantic canary	0	2	4	0	0.175	LC
*Spinus yarrellii* X *Serinus canaria*	Pintagol	2	0	2	0	0.100	-
Cardinalidae							
*Cyanoloxia brissonii* (Lichtenstein, 1823)	Ultramarine Grosbeak	9	0	21	7	0.925	LC
Icteridae							
*Gnorimopsar chopi* (Vieillot, 1819)	Chopi Blackbird	4	0	13	0	0.425	LC
*Icterus jamacaii* (Gmelin, 1788)	Campo Troupial	3	0	6	0	0.225	LC
Turdidae							
*Turdus rufiventris* (Vieillot, 1818)	Rufous-bellied Thrush	8	1	11	0	0.500	LC
Psittaformes							
Psittacidae							
*Amazona aestiva* (Linnaeus, 1758)	Turquoise-fronted Parrot	0	0	1	0	0.025	LC
*Aratinga auricapillus* (Kuhl, 1820)	Golden-capped Parakeet	0	0	1	0	0.025	NT

Recorded species are presented by taxonomic order and descending by the number of citations. The use value ($UV$) is calculated considering frequency of citation and attributed usages. Conservation status was defined by IUCN’s Red List.

### Bird-keeping

Motivations for keeping birds as pets were varied, ranging from attraction for the bird’s aesthetic, singing or the company, to statements that bird-keeping is a cultural tradition among local residents passed between generations. Urban residents that previously habited rural areas associated bird-keeping as a way to “remember the old home”, keeping species common to the previous inhabited place as a memento. Many of the interviewed (n = 26) also noted that among their local bird-keeping culture, there seems to be a preference for *Sporophila nigricollis* and *Saltator similis* individuals.

**Fig. 4:**
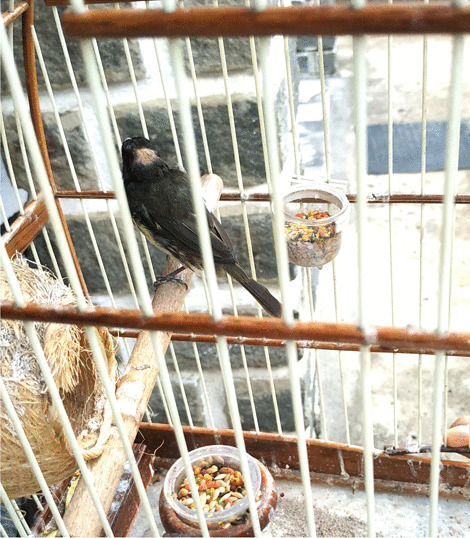
Captive *Sporophila nigricollis* displaying molting. In order to guarantee a healthy change of feathers, multiple types of food are provided

Respondents mentioned different practices to keep captive birds healthy. A common habit is to move bird cages to a sunny place every morning in order to guarantee enough natural light exposure to captive individuals. Different types of food were mentioned, varying between seeds of local Poaceae species to distinct rations, such as birdseed (“*alpiste*”) and millet (“*painço*”), for particular species. Captivity was reported to affect the bird’s plumage and singing. Periodic molting presented by some captive individuals is known as “feather recalling” by local bird-keepers, and is related foremost to the stress of captivity, especially in newly-caged individuals. Different approaches to decrease molting and increase the quality of plumage and singing were mentioned, including offering different types of seed, such as niger seeds (*Guizotia abyssinica* (L.f.) Cass*.*); fruits, such as papaya (*Carica papaya* L.) and oranges (*Citrus* × *sinensis* (L.) Osbeck); or applying vitamins to the captive individual (Fig. 4). Bird-keepers also can use the presence of other captive birds to encourage singing, a process described as “warming up”, done primarily by introducing a captive female of the species of the concerned individual in proximity. For territorial avian species, warming up can also be done by introducing another male in the vicinity.

### Bird trade

Commercialization of songbirds is regarded as a popular practice in the region and as a means to obtain money when needed. Some respondents admitted to participating in wild bird trade in the last year (n = 13), involving monetary compensation or using certain species as a currency in exchanges between other local known bird-keepers. Although the legal repercussion of these practices, such as apprehension of captive individuals and fining, are well-known facts among bird-keepers, all respondents that admitted engaging in bird trade stated that they do not see themselves as “bird traffickers”, justifying that in their own opinions, this would be a classification most suited to sellers of a large quantity of captive individuals involving a destination outside the city. As such, large scale bird trafficking is generally frowned upon by local bird-keepers, since it could attract surveillance’s attention to the region.

The interviewees reported that the value of traded birds depend on two main factors: the species and whether the captive bird was trained beforehand. Newly captured individuals (called by local bird-keepers as “wild”) were said to possess a much lower price, varying between U$ 5.00 to U$ 19.00 per individual according to species. After a process of “taming” which involves putting the wild bird in a small cage and familiarizing it with human presence, prices were said to vary between U$ 17.00 and U$ 114.00. This taming process could take between three months to one year, depending on the bird species and size (typically, larger birds would need more time to be tamed). Wild birds were said to be cheaper compared to tamed birds because newly captured birds could harm themselves against the cage in the first months of captivity, and would not sing as often or with the same attractiveness as tamed birds.

Trained songbirds that could perform distinct chants were said to be valued at much higher prices. *Sporophila nigricollis* individuals that could perform specific chants were said to be valued at prices as high as U$ 380.00, while the value of trained *Saltator similis* individuals could reach U$ 570.00. Training songbirds to perform specific chants was said to be a difficult and slow process that involved capturing wild individuals at a very young age and submitting them to hours of recordings of the desired chant, every day. Even with training, only a few individuals would be capable of performing the specific chant flawlessly. Territorial birds—such as *Cyanoloxia brissonii*—were said to be valued by “bravery”, as most aggressive birds would possess higher value in trade. The method to measure territorial birds’ aggressiveness was described as putting two encaged birds of the same species in the same vicinity; the first individual to bristle its feathers would be considered less “brave” than its counterpart. Trained birds were said to be submitted to compete against others of the same species in clandestine contests known as “*rinhas”*, often involving some kind of monetary prize. Songbirds would be tested against each other based on chant type or frequency, while territorial birds would compete by fighting against each other.

### Methods and strategies used to capture

**Fig. 5:**
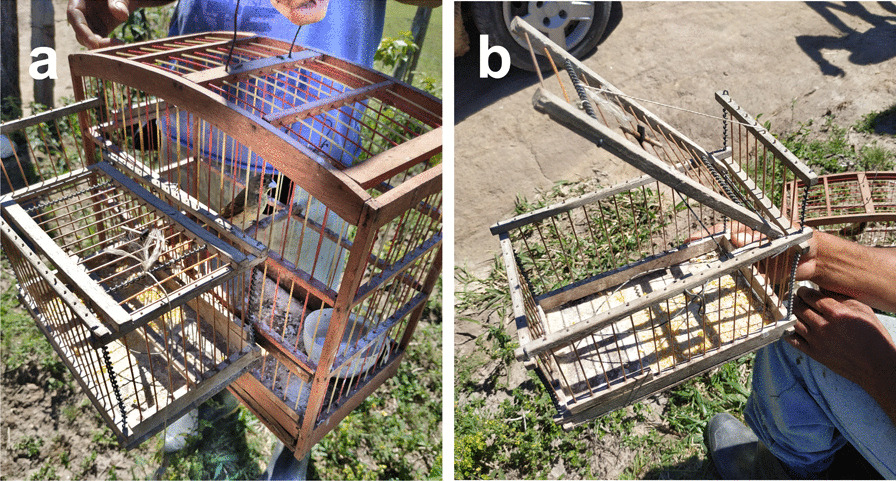
Tools used in bird trapping. Traps are often coupled with cages containing captive individuals of the desired species (**a**). In this picture, one of the most traditional traps to capture wild birds in the region, known as “alçapão” (**b**)

Most respondents reported capturing birds in the last year (n = 24), specifying this practice as a local tradition passed down through generations. Strategies reported to be used in capture would often involve specialized traps (Fig. 5) or procedures (Table 2) according to the desired species size, and would be performed at locations known for accommodating a large abundance of birds or the presence of particular species, often within the national park’s area. *Saltator similis*, for example, was said to be a species that could only be found in deep vine forest regions of the park and caught with traps suitable for larger birds, such as the “*esbarro*” or “*alçapão*”, while *Cyanoloxia brissonii* was mostly associated with the arid areas characterized as the Caatinga expanse of the park and would need a bait of the same species (labeled as a “*chama*”) to attract wild individuals to traps, as this is a territorial species.

**Table 2:** List of mentioned strategies used in bird trapping and frequency of citations of each

Method	Description	Used to catch	Number of citations
*Alçapão*	A cage-like trap made of wood, metal and fiber. It can only be opened on top. The main mechanism is set off by pressure; as the bird reaches the flooring of the trap, the mechanism goes off and lowers the upper lid, entrapping the bird	All types of avian species. Sometimes smaller species do not have enough weight to set off the closing mechanism and larger species cannot enter the trap, allowing those to escape	24
*Visgueira*	A type of natural glue extracted from jackfruit sap. It is typically applied in branches or twigs close to natural bodies of water. Often the glue can harm the entrapped bird when removing it from the trap	Smaller avian species. The glue is not strong enough to entrap larger species	12
*Esbarro*	A cage-like trap made of wood, metal and fiber. It can be opened at all sides, allowing birds coming from any direction to enter. As the bird touches the inside of the trap the main mechanism goes off, closing all sides of the trap simultaneously	All types of avian species, especially larger ones. Sometimes smaller species do not have enough weight to set off the closing mechanism, allowing those to escape	8
*Chama*	A captured bird that serves as bait to catch a particular species. Normally, it is located inside a small cage attached to a larger trap. It is especially effective to use a female of the desired species or a male when the desired species present territorial behavior	All types of avian species that can be attracted by other individuals from the same species or territorial species that tend to attack other avian species	5
Mirror	A piece of mirror located on the flooring of a trap that is set off by pressure, such as an *alçapão*. Seeing its own reflection in the mirror, the bird approaches the trap as if it was another individual	All types of avian species that can be attracted by other individuals from the same species or territorial species that tend to attack other avian species	2
Recorder	A recording of the desired species vocalization, usually reproduced by a smartphone nearby a trap	All types of avian species that can be attracted by another individual’s vocalization	1

Methods are displayed by the number of citations in descending order. All the names attributed to each method/trap, as well as their description and target species for each one was provided by interviewees who engage in bird trapping

The season also was said to influence avian species availability and capture. The optimal time for bird capture was said to be the rainy season, occurring between November and March. In this period, avian species abundance was said to increase next to natural bodies of water, such as waterfalls and rivers, and some popular species in the region, such as *Sporophila lineola*, could only be found and captured in this specific time period.

In the rural settlements, some respondents (n = 7) cited a practice known as “bird ordering”, a process that would involve the visit of an outsider offering advanced payments to local residents in exchange for captured avian species that they would later return to collect. Although no respondent admitted engaging in such practices, it was said that bird ordering is a recurring thing in the region, especially between known bird trappers and local large-scale traders. Another common occurrence is the visit of bird hunters from nearby cities to capture avian species located inside the park. Respondents linked those outsiders mostly to the cities of Manuel Vitorino (n = 29) and Poções (n = 27), areas where wild bird trade would be more present and even conducted in open markets through the cities.

### Bird-keeping and trade after the park’s establishment

**Fig. 6:**
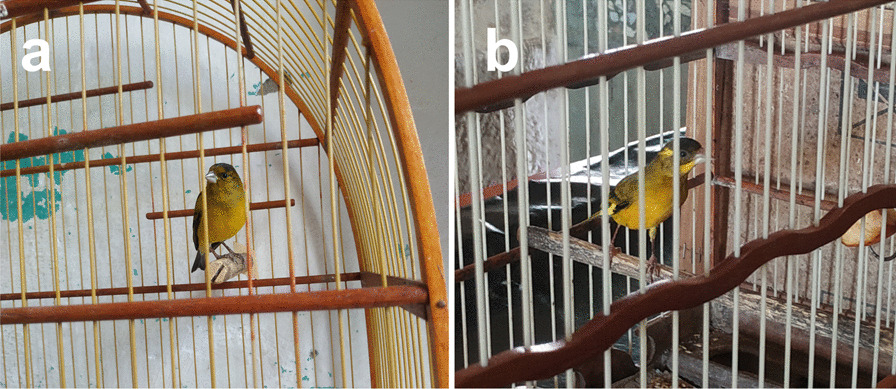
Hybrid individuals known locally as “Pintagol”. We identified both a juvenile (**a**) and an adult (**b**) individuals of this breeding between *Spinus yarrellii* and *Serinus canaria*

All respondents (n = 40) agreed that since the park’s establishment in 2010, practices of bird-keeping, capture and trade declined drastically within the urban areas of the city and rural settlements alike. The main reason attributed to this decline was the increased surveillance by environmental agencies, such as the Brazilian Institute of Environment and Renewable Natural Resources (IBAMA) and Chico Mendes Institute for Biodiversity’s Conservation (ICMBio), consequential to the park’s foundation. More than half of the urban areas’ respondents (n = 7) have had captive birds apprehended before by such agencies. In rural settlements, a few respondents (n = 4) admitted releasing captive birds in fear of penalty, after gaining knowledge that those agencies were inspecting their respective surroundings. Two respondents from urban areas explained that keeping a hybrid species obtained by breeding a male *Spinus yarrellii* with a female *Serinus canaria* could be a way of cheating surveillance by official agencies, and one of them could even breed those hybrids in his own home. Their reasoning was that as being hybrids, these individuals (Fig. 6) could not be reintroduced in a natural environment, therefore, surveillance agents would not make a significant effort to apprehend them.

## Discussion

The overall socioeconomic profile of interviewed bird-keepers and traders was consistent with what is generally described when discussing illegal bird trade in Brazil: low income and education levels, as well as inhabitants and occupations most related to rural settlements [21, 22, 45–47]. The prevalence of males is also recurrent among ethnological studies involving avian species [21, 45, 47–49] and seems to be a cultural factor associated with those practices, although there are exceptions [22, 46].

The Thraupidae family was shown to be the most popular among local bird-keepers, seeing that belonging species were most frequently cited as pets and in commercialization; especially those included in the *Sporophila* genus. Preference for the species of this particular family and genus is a recurring trend among bird-keepers [47]. A total of 21 avian species were cited; a relatively low number in comparison with other ethnological surveys [45, 48, 50, 51]. However, it is worth noting that with the exception of *Serinus canaria*—an exotic species—all cited species occur within the covered area of the park and its surroundings [31–33]. This number represents less than 5% of the total of estimated species (approximately 450) registered within the protected areas and Boa Nova’s surroundings [31–33, 43], an aspect that could be associated to the preference for songbirds and other ornamental species between bird-keepers, traders and trappers [5, 52]. Availability and access are also factors that may influence species preference for bird-keeping [23, 49], and could explain favoritism for certain species, seen that *Sporophila nigricollis* was the most popular species associated with bird-keeping and commercialization, even though other mentioned species such as *Sicalis flaveola*, *Paroaria Dominicana* and *Cyanoloxia brissoni* have considerable higher apprehension’s numbers in Brazil [52].

The main alleged way to obtain captive birds was through capture, a practice that seems to be common to both rural and urban settlements within the limits of Boa Nova’s municipality. Capturing avian species with the intention of keeping pets was said to be a local tradition passed forward by traditional knowledge, a tendency described in other areas alongside the Northeastern region [15, 49]. Many of the methods and traps mentioned by local residents are similar to other practices described in studies about bird trade and capture alongside the North and Northeastern regions, even though the analyzed region is considerably distant to those [46, 47, 51, 53]. Respondents also showed considerable knowledge about some species’ ecology, such as seasonal abundance fluctuations and preferred dwelling environments, yet another trait associated with areas where bird trapping and bird-keeping are considered cultural traditions [47]. Contrastingly, studies conducted within bigger city centers suggest commerce as the main source for captive bird’s acquisition [50].

Alongside capture, bird trade also seems to be a cultural habit among bird-keepers from the urban area, where many birds-keepers would maintain an informal contact network with other bird-keepers to buy, sell and trade avian species, using those as some sort of currency in clandestine exchanges. This kind of practice is easier to engage within urban settlements given the ease of access promoted by pavement roads and higher population density when compared to rural locations. The perceived dynamics of trade also share many traits with what is described in other ethnoornithological studies, such as this very trade chain between urban bird-keepers [45, 47] and the fact that most of the local traders do not see themselves as “bird traders” or “traffickers” but as hobbyists [51]. To them, the commercial aspect involved in bird-keeping practices would only be a part of collecting and raising avian species as pets. Pricing was also another factor that corresponded with the general descriptive trend, where newly caught birds would possess a much lower market value when compared to trained and tamed individuals [22, 45, 47]. In our study, we perceived a special demand for songbirds such as *Sporophila nigricollis* and *Saltator similis*. Individuals of these species who would excel at singing could reach prices ten times higher than their newly captured counterparts, according to local traders. The reasons for such an increase in value could be related to personal attraction for singing qualities or could be due to interest in submitting those songbirds in clandestine contests known as “*rinhas*”.

The demand for avian species that could be submitted for such contests is another recurring topic when discussing cultural aspects surrounding bird-keeping with Brazil [22, 47, 49, 51]. In this study, we identified two distinct types of contests: competitions involving songbirds—where those would be measured by singing qualities such as frequency, duration and volume of vocalization—and competitions involving physical fighting between territorial species. *Sporophila nigricollis* and *Saltator similis* were associated with singing contests, whereas *Cyanoloxia brissoni* was related only to bird fighting. Although *Paroaria domicana* and *Sicalis flaveola* were associated with bird-fighting in other studies [47, 49], there was no association between these species and bird-fighting by respondents, a factor that could suggest a regional trait regarding avian species preferences in contests.

Some bird-keepers displayed a specialized knowledge about some particular species’ nature and habits, such as the diet needed to raise a healthy individual in captivity. Some of the perceived customs and practices related to the maintenance of captive individuals were also observed by other authors, such as periodic molting and encouragement of singing by placing another captive individual in the vicinity [51]. One respondent even demonstrated specialized knowledge about how to breed *Spinus yarrellii* with *Serinus canaria*; the epithet received by these hybrid individuals (“*Pintagol*”) is the same as the one given by bird-keepers to hybrid individuals produced by breeding an *Spinus magellanicus* with *Serinus canaria* [53]; the reason for this association is most likely due to the similarity between the popular names of *Spinus magellanicus* (“*Pintassilgo-de-cabeça-preta*”) and *Spinus yarrellii* (“*Pintassilgo-do-nordeste*”) in Brazil. Although there are records of Passeriformes hybrids—such as *Spinus magellanicus* × *Serinus canaria*—associated to bird-keeping and bird trade in Brazil [54], this is the first register of a breeding between *Spinus yarrellii* and a *Serinus canaria*. Currently, it is not known if these hybrid individuals are fertile, although no breeding involving hybrids was reported. As stated by respondents who mentioned hybrid individuals, the usage of these as pets and in commercialization would be a way to evade surveillance by official environmental agencies. According to themselves, keeping hybrid individuals as pets would not constitute a crime, as the captive individual’s origin would be domestic, therefore, no individual would be removed from their natural habitat. It is worth noting that the Brazilian Federal Law Nº 9.605—responsible for disposing about environmental crimes and other related procedures—do not address the question of keeping hybrid individuals in captivity or submitting wild species to interspecies breeding, although some Brazilian states do criminalize such practices [54, 55]. Nevertheless, keeping *Spinus yarreli* individuals in captivity would constitute an environmental crime against fauna, as this is a native species in Brazil [56].

Since its establishment in 2010, the *Parque Nacional de Boa Nova* and its associated *Refúgio de Vida Silvestre de Boa Nova* have both been addressed in large operations regarding illegal wildlife trade surveillance and apprehension, with hundreds of captive birds being rescued [26, 29, 57]. It seems that large scale bird trade is acknowledged by local bird-keepers, and some of them shun this activity, as it would be a kind of endeavor maintained by a small number of known individuals within the city that would attract unwanted attention to all local birds-keepers from a legal perspective. All respondents agreed that after the protected area’s establishment and its consequential supervision by official agencies, the local tradition of bird-keeping and bird capture was heavily impaired; the main reason being the fear of apprehension and penalty. As such, many reported reducing the number of individuals maintained in captivity, freeing captive individuals or stopping bird-keeping altogether after apprehension. It is worth noting that many respondents mentioned habitants of nearby municipalities traveling in the park’s vicinity intending to capture wild birds. According to them, those bird trappers would avoid the main highways and travel by rural roads to dodge the attention of surveillance conducted in main highways, intending to sell newly captured birds within their own cities, where surveillance by environmental agencies would be less present or non-existent. Although the presented responses suggest a parallel and disconnected chain between local bird trade and large-scale wildlife trade occurring in the region, it should be noted that given the sensitive nature of the matter, many local bird trappers could be reluctant to admit any association or contact with large-scale bird traders. The knowledge about local large-scale traders and the dynamics of trade within the region displayed by many respondents would suggest some kind of, at least superficial, exchange between those who engage in bird-keeping and trapping as a cultural habit and large-scale traders responsible for gathering and directing a large number of captive birds to be sold in other locations.

Although some countries allow and regulate the use of wild species as pets at some extent, seeking to accomplish a sustainable exploitation of ornamental species without endangering local populations [5, 58]; in Brazil, any kind of unauthorized activity that involves captivity or any usage of wild species constitutes an environmental crime, including the unauthorized maintenance of wild species as pets [56]. Exploitation and transportation can impose significant disturbances on local populations, especially when particular individuals of a desired species are favored, as this could lead to population imbalances [59]; or when exotic species are released from captivity in environments where they do not occur naturally, a prospect that could proceed to ecological imbalances affecting natural populations [60]. Regarding avian species, population decline has been associated with trapping and illegal commercialization globally, with situations ranging from a decrease in local populations most vulnerable to selective hunting and capture [10], to an overall global population decline of migratory avian species chased by local bird trappers and hunters [11]. Considering such consequences, some authors suggest law implementation and enforcement as one the most effective ways to protect local avian populations [23, 61, 62].

Some of the mentioned species are facing threatened conservations status according to IUCN’s Red List: *Aratinga auricapillus* is a Psittacidae species occurring in ecosystems associated to the Atlantic Forest that is currently facing a Near Threatened conservation status, attributed mainly to habitat loss marked by fragmentation, as well as entrapment for illegal trade [44, 63]. *Sporophila falcirostris* is a nomadic species specialized in using bamboo seeds as a food source who is currently under a Vulnerable conservation status due habitat degradation, as well as illegal trapping for commercialization [44, 64]. *Spinus yarrelii’*s populations are suspected to be rapidly declining, as this species is currently under a Vulnerable conservation status attributed mainly to the illegal wild bird trade [43, 44]. Lastly, *Sporophila maximiliani* is considered an Endangered species due to its small populations that are declining as a result of habitat degradation caused by agriculture, as well as intensive trapping for bird-keeping and bird trade [44, 65]. It is worth noting that the above-mentioned species scored some of the lowest $UVs$ recorded in this study (with exception of *Spinus yarrellii*, due to it being one of the few species associated with breeding). This fact could be related to a lower availability, considering the decline of local populations globally, or simply an overall lower interest in such species to use in commercialization or as pets. The highest recorded $UVs$ refer to species presenting a Least Concern conservation status: *Sporophila nigricollis* occurs at an extremely large range, and its populations appear to be growing as habitat degradation creates additional areas of suitable habitat [44, 66]. *Saltator similis* also occur at a large geographical area, even though their populations seem to be declining at a steady, slower pace [44, 66]. Population trends for *Cyanoloxia brissonii* are currently unknown, however, it is not believed that decline is occurring promptly enough to uphold a threatened conservation status, and this species has a considerable large range of occurrence as well [44, 66]. Although these associations could point to a relationship between $UVs$ and availability, as exploitation would be expected to be halted according to the local decline of native populations, it should be noted that rarity is often a driver behind the demand and popularity of certain animal products, composing a human-generated feedback loop that can accentuate the decline of already small and decreasing populations [67, 68]. Therefore, $UVs$ should not be used as a reliable metric to appraise the conservation status of local populations.

## Conclusion

Bird-keeping and related practices are cultural habits among local communities surrounding the *Parque Nacional de Boa Nova*. We observed many customs and habits similar to what is described regarding bird-keeping in Brazil, as well as some novelty practices, such as interspecies breeding in order to obtain hybrid individuals. Local preferences for particular species are in accordance with what is commonly reported in the associated literature, although access to species that occur through the protected areas seems to influence some local particularities regarding preferences and uses. Although bird-keeping and bird capture seems to be present in both urban and rural settlements within Boa Nova, the dynamics involved in bird trade seem to occur mainly in urban areas, where bird-keepers who seem themselves as hobbyists conduct a local trade chain involving mostly trained captive birds, with each other. The establishment of the protected areas seemed to greatly impact local community’s perceptions about bird-keeping and bird trade in general, mainly through fear of apprehension and penalty for keeping native species in captivity, as a consequence from the increased surveillance from environmental agencies responsible for the park’s management. Although this kind of perception is constructive for conservation in the short-term, some individuals will search for ways to circumvent surveillance, such as releasing captive individuals when they know surveillance is close, or even keeping hybrid species as pets. As such, it is imperative for long-term conservation to maintain a socioenvironmental balance between local communities and protected areas. For this to happen, it would be necessary to find legal ways to integrate local bird-keepers in the conservation of local species, such as promoting the creation of sustainable collection reserves of local species according to their conservation status, under established regulations. Otherwise, educational and awareness actions against animal abuse and captivity could be implemented as a way to mitigate the demand for native avian species in the first place. We recommend further studies about the effectiveness of these kinds of approaches on a local community’s ethnoornithological perception.

## Data Availability

The datasets generated and/or analyzed during the current study are not publicly available, as personal information regarding location, gender, age and income could reveal the identity of respondents, breaching the anonymity guaranteed when agreeing to participate in this research by the researchers and the ethics committee. Any kind of data that would not breach the respondent’s anonymity can be obtained from the corresponding author under reasonable request.

## Abbreviations

CAAE: Certificate of Presentation for Ethical Consideration
IUCN: International Union for Conservation of Nature
UV: Use value
MCA: Multiple Correspondence Analysis
IBAMA: Brazilian Institute of Environment and Renewable Natural Resources
ICMBio: Chico Mendes Institute for Biodiversity’s Conservation

## References

[1] Baker SE, Cain R, van Kesteren F, Zommers ZA, D’Cruze N, Macdonald DW (2013). Rough trade: animal welfare in the global wildlife trade. Bioscience.

[2] Barber-Meyer SM (2010). Dealing with the clandestine nature of wildlife-trade market surveys. Conserv Biol.

[3] dos Santos LP, de Araujo DR (2015). Aspectos socioeconômicos dos municípios Brasileiros com ocorrência de tráfico de animais silvestres no bioma Cerrado. Élisée Rev Geogr UEG.

[4] Wyler LS, Sheikh PA. International illegal trade in wildlife: threats and U.S. Policy. Libr Congr Wash DC Congr Res Serv. 2008. https://sgp.fas.org/crs/misc/RL34395.pdf. Accessed 07 Out 2021.

[5] Roldán-Clarà B, Lopez-Medellín X, Espejel I, Arellano E (2014). Literature review of the use of birds as pets in Latin-America, with a detailed perspective on Mexico. Ethnobiol Conserv.

[6] Bush ER, Baker SE, Macdonald DW (2014). Global trade in exotic pets 2006–2012. Conserv Biol J Soc Conserv Biol.

[7] Roldán-Clarà B, López-Medellín X, de la Barca NC, Leyva C, Espejel I (2017). Mexican birds use according to environmental officers. Ethnobiol Conserv.

[8] Nascimento CA, Czaban R, Alves R (2015). Trends in illegal trade of wild birds in Amazonas State, Brazil. Trop Conserv Sci.

[9] Leupen B, Gomez L, Shepherd C, Nekaris KA, Imron M, Nijman V (2020). Thirty years of trade data suggests population declines in a once common songbird in Indonesia. Eur J Wildl Res.

[10] Williams VL, Cunningham AB, Kemp AC, Bruyns RK (2014). Risks to birds traded for African traditional medicine: a quantitative assessment. PLoS ONE.

[11] Kamp J, Oppel S, Ananin AA, Durnev YA, Gashev SN, Hölzel N (2015). Global population collapse in a superabundant migratory bird and illegal trapping in China. Conserv Biol.

[12] Hernandez ÉFT, de Carvalho MS (2006). O tráfico de animais silvestres no Estado do Paraná. Acta Sci Hum Soc Sci.

[13] Bezerra DMM, de Araujo HFP, Alves ÂGC, Alves RRN (2013). Birds and people in semiarid northeastern Brazil: symbolic and medicinal relationships. J Ethnobiol Ethnomed.

[14] dos Santos Soares VM, de Lucena Soares HK, da Silva SS, de Lucena RFP (2018). Local knowledge, use, and conservation of wild birds in the semi-arid region of Paraíba state, northeastern Brazil. J Ethnobiol Ethnomedicine.

[15] Teixeira PHR, Thel TN, Ferreira JMR, de Azevedo SM, Junior WRT, Lyra-Neves RM (2014). Local knowledge and exploitation of the avian fauna by a rural community in the semi-arid zone of northeastern Brazil. J Ethnobiol Ethnomed.

[16] Destro GFG, de Marco P, Terribile LC (2020). Comparing environmental and socioeconomic drivers of illegal capture of wild birds in Brazil. Environ Conserv.

[17] Destro GFG, Lucena T, Monti R, Cabral R, Barreto R. Efforts to combat wild animals trafficking in Brazil. In: Lameed GA (ed.) Biodiversity Enrichment in a diverse world. Intechopen. 2012.

[18] Giovanini D. 1° Relatório Nacional sobre o Tráfico de Fauna Silvestre. RENCTAS. 2002. https://www.renctas.org.br/wpcontent/uploads/2014/02/REL_RENCTAS_pt_final.pdf. Accessed 07 Out 2021.

[19] de Souza GM, Oliveira A (2005). O Comércio Ilegal De Aves Silvestres Na Região Do Paraguaçu E Sudoeste Da Bahia. Enciclopédia Biosf.

[20] Gama TP, Sassi R (2008). Aspectos do comércio ilegal de pássaros silvestres na cidade de João Pessoa, Paraíba. Brasil Gaia Sci.

[21] Rocha MSP, Cavalcanti PCM, Sousa RL, Alves RRN (2006). Aspectos da comercialização ilegal de aves nas feiras livres de Campina Grande, Paraíba. Brasil Rev Biol E Ciênc Terra.

[22] Alves RRN, Nogueira EEG, Araujo HFP, Brooks SE (2010). Bird-keeping in the Caatinga. NE Brazil Hum Ecol.

[23] Alves RRN, Lima JRF, Araujo HFP (2013). The live bird trade in Brazil and its conservation implications: an overview. Bird Conserv Int.

[24] Tebaldi ALC, Fiedler NC, Dias HM (2012). Vulnerability and management of protected areas from the state of Espirito Santo, Brazil. FLORAM.

[25] Alves RRN, Souto WM (2011). Ethnozoology in Brazil: current status and perspectives. J Ethnobiol Ethnomedicine.

[26] Correio. MPF denuncia associação criminosa especializada em tráfico de animais silvestres, no Parque Nacional de Boa Nova. 2021. https://www.correio24horas.com.br/noticia/nid/mpf-denuncia-associacao-criminosa-em-trafico-de-animais-silvestres-na-bahia/. Accessed 07 Out 2021.

[27] Agência Sertão. PF deflagra operação de combate ao tráfico de pássaros silvestres no sudoeste baiano. 2020. https://agenciasertao.com/2020/08/27/pf-deflagra-operacao-de-combate-ao-trafico-de-passaros-silvestres-no-sudoeste-baiano/. Accessed 07 Out 2021.

[28] G1. Fiscalização apreende mais de 170 aves em condições de maus-tratos dentro de veículo no sudoeste da Bahia. 2021. https://g1.globo.com/ba/bahia/noticia/2021/03/24/fiscalizacao-apreende-mais-de-170-aves-em-condicoes-de-maus-tratos-dentro-de-veiculo-no-sudoeste-da-bahia.ghtml. Accessed 07 Out 2021.

[29] Tavares S. Boa Nova é destino para observadores de aves. Instituto Chico Mendes de Conservação da Biodiversidade. 2015. https://www.icmbio.gov.br/portal/ultimas-noticias/20-geral/7111-boa-nova-e-destino-para-observadores-de-aves. Accessed 07 Out 2021.

[30] IBGE. Cadastro Central de Empresas. 2019. https://biblioteca.ibge.gov.br/visualizacao/livros/liv101720.pdf. Accessed 07 Out 2021.

[31] Ebird. eBird: An online database of bird distribution and abundance. EBird Cornell Lab Ornithol. 2021.

[32] Wikiaves. Parque Nacional de Boa Nova. 2021. https://www.wikiaves.com.br/wiki/areas:pn_de_boa_nova:inicio. Accessed 07 Out 2021.

[33] Wikiaves. Refúgio de Vida Silvestre de Boa Nova. 2021. https://www.wikiaves.com.br/wiki/areas:rvs_de_boa_nova:inicio. Accessed 07 Out 2021.

[34] Brasil. DECRETO DE 11 DE JUNHO DE 2010. MINISTÉRIO DO MEIO AMBIENTE - MMA. 2010.

[35] da Silva TLL, Moura JMB, Hora JSL, de Oliveira ES, dos Souza AS, da Silva NA, de Lucena RFP, da CruzCunha LVF, Alves RRN (2019). Implementing ethnobiological research: pretests, quality control, and protocol reviews. Methods and techniques in ethnobiology and ethnoecology.

[36] Noy C (2008). Sampling knowledge: the hermeneutics of snowball sampling in qualitative research. Int J Soc Res Methodol.

[37] de Sousa DCP, Magalhães HF, de Oliveira ES, Albuquerque UP, de Lucena RFP, da Cruz Cunha LVF, Alves RRN (2019). Qualitative data analysis. Methods and techniques in ethnobiology and ethnoecology.

[38] Albuquerque U, Lucena R, Monteiro J, Nunes A, Almeida C (2006). Evaluating two quantitative ethnobotanical techniques. Ethnobot Res Appl.

[39] Husson F, Lê S, Pagès J (2011). Exploratory multivariate analysis by example using R.

[40] Grantsau R, Júnior P. Guia completo para identificação das aves do Brasil. Vento Verde Editora; 2010.

[41] Pacheco JF, Silveira LF, Aleixo A, Agne CE, Bencke GA, Bravo GA (2021). Lista comentada das aves do Brasil pelo Comitê Brasileiro de Registros Ornitológicos. Rev Bras Ornitol.

[42] Endrigo E. Aves da Mata Atlântica. Aves & Fotos Editora; 2006.

[43] Gonzaga LP, Pacheco JF, Bauer C, Castiglioni GDA (1995). An avifaunal survey of the vanishing montane Atlantic forest of southern Bahia, Brazil. Bird Conserv Int.

[44] IUCN. The IUCN Red List of Threatened Species. 2021. https://www.iucnredlist.org/. Accessed 07 Out 2021.

[45] de Oliveira WSL, Borges AKM, de Faria LS, Vasconcellos A, Alves RRN (2020). Illegal trade of songbirds: an analysis of the activity in an area of northeast Brazil. J Ethnobiol Ethnomedicine.

[46] Barbosa JAA, Nobrega VA, Alves RRN (2010). Aspectos da caça e comércio ilegal da avifauna silvestre por populações tradicionais do semi-árido paraibano. Rev Biol E Ciênc Terra.

[47] Souto WMS, Torres MAR, Sousa BFCF, Lima KGGC, Vieira LTS, Pereira GA (2017). Singing for cages: the use and trade of passeriformes as wild pets in an economic center of the Amazon—NE Brazil Route. Trop Conserv Sci.

[48] Alves RRN, Leite RCL, Souto WMS, Bezerra DMM, Loures-Ribeiro A (2013). Ethno-ornithology and conservation of wild birds in the semi-arid Caatinga of northeastern Brazil. J Ethnobiol Ethnomedicine.

[49] Bezerra MMD, Araujo FPH, Alves RRN (2017). Keeping wild birds as pets in a semiarid region of rio grande do norte state, northeastern brazil. Hornero.

[50] Licarião MR, Bezerra DMM, Alves RRN (2013). Wild birds as pets in Campina Grande, Paraíba State, Brazil: an ethnozoological approach. An Acad Bras Ciênc.

[51] Fernandes-Ferreira H, Mendonça SV, Albano C, Ferreira FS, Alves RRN (2012). Hunting, use and conservation of birds in Northeast Brazil. Biodivers Conserv.

[52] Costa FJV, Ribeiro RE, de Souza CA, Navarro RD (2018). Espécies de Aves Traficadas no Brasil. Front J Soc Technol Environ Sci.

[53] Bezerra DMM, de Araujo HFP, Alves RRN (2012). Captura de aves silvestres no semiárido brasileiro: técnicas cinegéticas e implicações para conservação. Trop Conserv Sci.

[54] Eduardo Leite. Decreto N^o^ 55374 DE 22/01/2020. 2020. https://www.legisweb.com.br/legislacao/?id=398911#:~:text=Das%20Disposi%C3%A7%C3%B5es%20Gerais-,Art.,do%20Sul%2C%20e%20os%20arts. Accessed 07 Out 2021.

[55] Assembleia Legislativa do Estado de São Paulo. Lei n^o^ 16.784, de 28 de junho de 2018. 2018. https://www.al.sp.gov.br/repositorio/legislacao/lei/2018/lei-16784-28.06.2018.html#:~:text=Pro%C3%ADbe%20a%20ca%C3%A7a%20no%20Estado%20de%20S%C3%A3o%20Paulo%20e%20d%C3%A1%20outras%20provid%C3%AAncias. Accessed 07 Out 2021.

[56] Brasil. LEI N^o^ 9.605, DE 12 DE FEVEREIRO DE 1998. Presidência da República. 1998. http://www.planalto.gov.br/ccivil_03/leis/l9605.htm#:~:text=L9605&text=LEI%20N%C2%BA%209.605%2C%20DE%2012%20DE%20FEVEREIRO%20DE%201998.&text=Disp%C3%B5e%20sobre%20as%20san%C3%A7%C3%B5es%20penais,ambiente%2C%20e%20d%C3%A1%20outras%20provid%C3%AAncias. Accessed 07 Out 2021.

[57] O Norte. Polícia Federal desarticula esquema de tráfico de aves em MOC e outras 11 cidades do Estado. 2020. https://onorte.net/minas-do-norte/pol%C3%ADcia-federal-desarticula-esquema-de-tr%C3%A1fico-de-aves-em-moc-e-outras-11-cidades-do-estado-1.801509. Accessed 07 Out 2021.

[58] Roldán-Clarà B, Toledo VM, Espejel I (2017). The use of birds as pets in Mexico. J Ethnobiol Ethnomed.

[59] Webb J, Brook B, Shine R (2002). Collectors endanger Australia’s most threatened snake, the broad-headed snake Hoplocephalus bungaroides. Oryx.

[60] Yosef R, Zduniak P, Żmihorski M (2016). Invasive ring-necked parakeet negatively affects indigenous Eurasian Hoopoe. Ann Zool Fenn.

[61] Lee RJ, Gorog AJ, Dwiyahreni A, Siwu S, Riley J, Alexander H (2005). Wildlife trade and implications for law enforcement in Indonesia: a case study from North Sulawesi. Biol Conserv.

[62] Herrera M, Hennessey B (2007). Quantifying the illegal parrot trade in Santa Cruz de la Sierra, Bolivia, with emphasis on threatened species. Bird Conserv Int.

[63] Cordeiro PHC. A Fragmentação da Mata Atlântica no Sul da Bahia e suas Implicações na Conservação dos Psitacídeos. In: Corredor De Biodiversidade Da Mata Atlântica Do Sul Da Bahia. Ornis Meio Ambiente e Desenvolvimento. 2003. https://ibama.angelfire.com/papagaios.pdf. Accessed in 07 Out 2021.

[64] Areta J, Bodrati A, Thom G, Eisen Rupp A, Velazquez M, Holzmann I (2013). Natural history, distribution, and conservation of two nomadic Sporophila seedeaters specializing on bamboo in the Atlantic Forest. Condor.

[65] ICMBIO. Livro Vermelho da Fauna Brasileira Ameaçada de Extinção. 2018. https://www.icmbio.gov.br/portal/images/stories/comunicacao/publicacoes/publicacoes-diversas/livro_vermelho_2018_vol1.pdf. Accessed in 07 Out 2021.

[66] Stotz DF (1996). Neotropical birds: ecology and conservation.

[67] Aisher A (2016). Scarcity, alterity and value: decline of the pangolin, the world’s most trafficked mammal. Conserv Soc.

[68] Courchamp F, Angulo E, Rivalan P, Hall RJ, Signoret L, Bull L (2006). Rarity value and species extinction: the anthropogenic allee effect. PLoS Biol.

